# persistent pain and fatigue drive reduced quality of life in treated Gaucher disease type 1: a cross-sectional analysis

**DOI:** 10.1186/s13023-026-04452-w

**Published:** 2026-06-16

**Authors:** Shauna L. Mangum, Stephen H. A. Hernandez, Elizabeth L. Dickson, Wendy Packman, Beth Tigges

**Affiliations:** 1 Edson College of Nursing and Health Innovation, Arizona State University, 550 N. 3rd St., Mail Code 3020, Phoenix, AZ 85032 USA; 2 College of Nursing, University of Texas El Paso, 1851 Wiggins Rd, El Paso, TX 79968 USA; 3 College of Population Health, University of New Mexico, 2300 Tucker Ave NE, Albuquerque, NM 87131 USA; 4 Palo Alto University, 1791 Arastradero Rd, Palo Alto, CA USA; 5 College of Nursing, University of New Mexico, 2300 Tucker Rd. Building 214, NE, Albuquerque, NM 87131 USA

**Keywords:** Fatigue, Gaucher disease, Health related quality of life (HRQoL), Pain, Rare disease, Symptom

## Abstract

**Purpose:**

Gaucher disease type 1 (GD1) is a multisystem lysosomal disorder in which treatment with enzyme replacement therapy (ERT) or substrate reduction therapy (SRT) improves key clinical parameters. However, many patients continue to experience persistent symptoms that are not captured by traditional disease markers. This study evaluated the relationship between pain, fatigue, and health-related quality of life (HRQoL) among treated adults with GD1.

**Methods:**

In this cross-sectional study, adults with GD1 receiving ERT or SRT (*N* = 97) completed an online survey including the 36-item Short Form Survey (SF-36), PROMIS^®^ Fatigue and Pain Interference Short Forms, and the Treatment Satisfaction Questionnaire for Medication©. Associations between demographic factors, treatment variables, symptoms, and HRQoL were assessed using correlation analyses, t-tests, ANOVA, and multiple regression.

**Results:**

Pain and fatigue were strong, independent predictors of both physical and mental HRQoL, with age additionally predicting physical HRQoL. Significant negative correlations were observed between pain and HRQoL (physical and mental), as well as between fatigue and HRQoL. Participants reported significantly lower HRQoL compared with U.S. population norms across 7 of 8 SF-36 domains.

**Conclusions:**

Persistent pain and fatigue represent major, under-recognized drivers of impaired quality of life in treated GD1, indicating that current therapies do not fully address the symptomatic burden of disease. These findings support routine integration of validated patient-reported outcome measures into clinical care and highlight the need for adjunctive and mechanism-based approaches to improve patient-centered outcomes.

## Introduction

Gaucher disease is a rare, autosomal recessive disorder caused by a deficiency of the lysosomal enzyme, glucocerebrosidase [1] It is classified into three broad phenotypes [2]. Gaucher disease type 1 (GD1) is characterized by the absence of neurodegenerative disease while Gaucher disease type 2 (acute neuronopathic) and type 3 (chronic neuronopathic) are defined by the presence and severity of neurodegenerative disease. GD1 is estimated to affect approximately 1 in 40,000 to 1 in 60,000 individuals in the general population, with much higher incidence among people of Ashkenazi Jewish descent, approximately 1 in 850 [3]. Approximately 6,000 people are currently living in with GD1 in the United States [4].

The clinical manifestations of GD1 arise from reduced glucocerebrosidase activity, leading to accumulation of its substrate, glucosylceramide, within macrophages and the formation of characteristic Gaucher cells. These cells accumulate primarily in the bone marrow, spleen, and liver, but may also infiltrate other organs, including the lungs [5]. This process results in a wide spectrum of clinical features, including anemia, thrombocytopenia, hepatosplenomegaly, pulmonary involvement, immune dysregulation, and skeletal disease. Skeletal manifestations—such as chronic bone pain, osteopenia, osteoporosis, avascular necrosis, medullary infarction, and pathologic fractures—are particularly debilitating and represent major contributors to reduced health-related quality of life (HRQoL) [2]. Pain and fatigue are among the most commonly reported symptoms in GD1. Pain is often attributed to skeletal involvement and affects the majority of patients [6, 7]. In addition, abdominal discomfort may arise from hepatosplenomegaly [8]. Fatigue, although frequently reported, remains less well characterized in GD1. Importantly, fatigue does not consistently correlate with traditional clinical measures and may reflect a complex interplay of hematologic abnormalities, organ involvement, skeletal disease, and inflammatory or immune-mediated processes [9–11]. Together, these symptoms contribute substantially to impaired HRQoL.

There are currently two types of treatment available for GD1 [1], each with a distinct mechanism of action: Enzyme Replacement Therapy (ERT) and Substrate reduction therapy (SRT) [12]. ERT is an intravenous treatment that supplies the missing enzyme, glucocerebrosidase, which is taken up by macrophages and transferred to lysosomes [1]. Currently, three ERTs are available: Imiglucerase, velaglucerase, & taliglucerase alfa. Individuals receiving ERT must commit to intravenous enzyme replacement infusions every two to four weeks, a procedure that can be both painful and time-consuming [13]. SRT, in contrast, minimizes the accumulation of glucosylceramide production in the cell, which makes the residual glucocerebrosidase activity sufficient [14]. Unlike ERT, SRT is administered orally, requiring one to three daily doses. Research has demonstrated that both ERT and SRT result in improved clinical markers including increased platelet count and hemoglobin concentration, improved biomarkers, reduced liver and spleen volumes, and improved skeletal pathology [1, 15–17]. However, despite these advances, many patients continue to report persistent symptoms such as pain and fatigue, suggesting that current therapies may not fully address the overall disease burden.

## Summary of literature

Prior to treatment availability, HRQoL among adults with GD1 was lower than U.S. population norms. Studies consistently demonstrate HRQoL improvements following treatment initiation [18–24]. Similar patterns have been observed in pediatric and younger populations as well [25]. Research examining sex differences in HRQoL among adults with GD1 is limited, however, prior research reported that females initiating ERT (imiglucerase) were 4.5 times more likely than males (95% CI 2.19–9.25) to report improvements in HRQoL in the General Health subscale from the SF-36 survey, and 2.33 (95% CI 1.30–4.18) times more likely to report improvements in the Physical Health subscale, although overall HRQoL was still lower than the general population [18]. More recent available evidence suggests that females report lower HRQoL than males despite being on treatment [20, 26].

The homozygous N370S/N370S genotype is associated with milder manifestations of GD1 compared to more severe disease with the N370S/L444P genotype [26–28]. Findings on the relationship between genotype and HRQoL have been mixed. In one study, it was found that individuals with homozygous N370S/N370S reported higher HRQoL compared to other genotypes [23], however there was no difference in HRQoL across genotypes in a separate study [19]. Increasing age has been associated with lower HRQoL among adults with GD1 [19, 29].

Treatment has been shown to reduce pain with both ERT [30, 31] and SRT [32]. Fatigue has also been reported to improve with ERT [21, 33] and either improve or remain stable with SRT [15, 34–35]. However, despite these treatment-related improvements, a substantial proportion of patients continue to report persistent symptoms, suggesting that gains in traditional clinical measures may not fully translate into normalization of HRQoL.

### Why this study was necessary

There have been substantial advances in therapeutic options for rare genetic diseases, including GD1 [36]. Although people with GD1 have had access to ERT and SRT for many years, and these treatments have been shown to improve HRQoL, many long-term treated individuals continue to experience persistent symptoms and reduced quality of life.

Even when ERT and SRT improve clinical features such as liver and spleen volume and platelet counts, studies demonstrate that symptoms such as pain and fatigue may persist and negatively affect HRQoL despite long-term treatment [9, 15, 16, 46]. This disconnect suggests that traditional clinical markers may not fully capture the overall disease burden experienced by patients.

Accordingly, there is a need to better characterize the relationship between patient-reported symptoms and HRQoL in treated GD1. This study begins to address this gap by examining the broad associations between pain, fatigue, and HRQoL, with the future goal of informing more comprehensive and patient-centered approaches to care.

### Purpose

The purpose of this cross-sectional study was to examine the relationships between individual characteristics, symptoms, and health-related quality of life (HRQoL) among adults with GD1. Specifically, the study addressed the following research questions:

- Do individual characteristics (sex, race, ethnicity, and genotype) relate to HRQoL among individuals with GD1?
- Are individual characteristics, specifically sex and genotype, associated with differences in pain and fatigue among individuals with GD1?
- Are pain and fatigue associated with HRQoL among individuals with GD1?
- Does age, treatment type (ERT or SRT), treatment length, treatment satisfaction, pain, and fatigue independently predict HRQoL among individuals with GD1?

## Methods

### Design and setting

This study used a cross-sectional, self-administered survey design. Eligible participants were recruited from the Gaucher Community Alliance© (GCA), National Gaucher Foundation© (NGF), International Gaucher Alliance© (IGA), National Gaucher Foundation of Canada© (NGFC), Gaucher treatment centers, and the Gaucher online networking listserv website at Groups.io. The invitation was posted on respective websites and social media accounts. Additionally, a letter describing the purpose of the study with the survey link was emailed to Gaucher treatment centers to forward to patients with GD1so those who were interested in participating in this research could access the link. Participants were asked to complete a secure, anonymous electronic Research Electronic Data Capture (REDCap™) survey [37–38] hosted by the University of New Mexico Health Sciences Center.

### Characteristics of participants

Participants included 97 adults (age 18 and over) with a confirmed diagnosis of GD1 who were currently taking ERT or SRT and who could read and understand English. Participants were excluded if they were under 18, were not receiving any treatment or were taking both treatments concurrently.

### Measures

The final 76-item survey included (1) an individual characteristic questionnaire (age, sex, race, ethnicity, Gaucher disease genotype, type of treatment, length of time on treatment); (2) the 36-item Short Form Survey (SF-36) for HRQoL and its two summary measures of Physical Component Summary (PCS) and Mental Component Summary (MCS), each with standardized scores ranging from 0 (worst) -100 (best) [39–42]. The PCS is made up of four SF-36 subscales including physical functioning, role limitations due to physical functioning, bodily pain, and general health. The MCS is made up of four SF-36 subscales including vitality, social functioning, role limitations due to emotional problems, and mental health; (3) the 3-item Global Satisfaction Subscale of the Treatment Satisfaction Questionnaire for Medication (TSQM©); [43] with scores ranging from 0 to 100 and higher scores indicating more satisfaction; (4) the 8-item PROMIS^®^ Item Bank v1.0 – Fatigue – Short Form 8a, and (5) the 8-item PROMIS^®^ Item Bank v1.1 – Pain Interference – Short Form 8a [44]. Higher T-scores on both PROMIS^®^ measures indicate greater pain and fatigue (T-score ranges = 0–100) for each measure.

### Statistical analysis

IBM^®^ SPSS^®^ Statistics (v. 29) was used for the statistical analysis. Individual sample t-tests, one-way and two-way between groups ANOVA, and Pearson r correlation were used to determine differences and associations between demographic and symptom variables and HRQoL Multiple linear regression analyses were conducted to determine if demographic variables, pain, and fatigue predict physical and mental HRQoL. A post hoc power analysis conducted using G*Power version 3.1.9.4 [45] demonstrated a power of 0.80 to detect a medium effect size (Cohen’s *f2*=0.15) with the sample of 97, linear multiple regression with six predictors, and a significance level of α = 0.05.

## Results

A total of 97 adults with GD1 responded to the survey (Table 1). Most respondents were female (73%), non-Hispanic (92.3%), with an average age of 55.4 years. There were seven respondents (7.7%) who identified as Hispanic or Latino ethnicity and identified their race as White. Half of the respondents were homozygous for the N370S mutation. Two thirds of the participants received ERT, while the remainder one-third received SRT.

**Table 1:** Characteristics of study participants (*N* = 97)

Individual Characteristics	*n*	%
Age		
18–24 years	4	4.1
25–34 years	6	6.2
35–44 years	13	13.4
45–54 years	16	16.5
55–64 years	16	16.5
65 years and over	29	29.9
Sex		
Male	24	27
Female	65	73
Ethnicity		
Hispanic or Latino	7	7.7
Non-Hispanic or Latino	84	92.3
Race		
Black or African American	1	1.1
White	87	92.6
Genotype		
N370S/N370S	38	49.4
N370S/L444P	18	23.4
N370S/84GG	1	1.3
Other	16	20.8
Unknown	4	5.1
Type of Treatment		
ERT	64	67.4
imiglucerase	31	48.4
taliglucerase alfa	6	9.4
velaglucerase alfa	27	42.2
SRT	31	32.6
eliglustat	30	96.8
miglustat	1	3.2
Length of time on current treatment		
0–5 years	18	20
6–10 years	24	25.3
11–15 years	13	13.7
16–20 years	9	9.5
21–25 years	5	5.3
26 years or more	25	26.3
Length of time on any treatment		
0–5 years	8	8.4
6–10 years	6	6.3
11–15 years	18	6.3
16–20 years	12	12.6
21–25 years	15	15.8
26 years or more	36	37.9

### Health-related quality of life by demographics, treatment, and genotype

Independent samples *t*-tests (Table 2) indicated that males had slightly higher physical (PCS) and mental (MCS) HRQoL than females, but differences were not significant. HRQoL did not differ by race/ethnicity. Physical HRQoL was higher among individuals on SRT, though not significant, and treatment type was unrelated to mental HRQoL. ANOVA results indicated that adults with N370S homozygous genotype had higher physical and mental HRQoL, although these differences were not significant: PCS *F* (2, 74) = 0.38, *p* = .69, *η*^*2*^ = 0.01; MCS *F* (2, 74) = 1.41, *p* = .25, *η*^*2*^ = 0.04. Table 3 shows means and standard deviations for all genotype groups.

**Table 2:** Independent samples t-tests, means and standard deviations for sex, race/ethnicity, and treatment type and physical (PCS) and mental (MCS) HRQoL

Demographics	PCS					MCS				
	M	SD	t	*p*	Cohen’s d	M	SD	t	*p*	Cohen’s d
Sex			*t*(87)0.77	0.44	0.17			*t*(87)0.77	0.45	0.16
Male	68.13	20.42				70.15	17.71			
Female	63.74	24.88				66.10	23.95			
Race/Ethnicity			*t*(85)-0.21	0.83	0.05			*t*(85)-0.30	0.76	0.07
White, non- Hispanic	63.77	23.74				66.29	22.18			
Hispanic	65.80	29.30				69.02	29.21			
Treatment Type			t(93)1.80	0.08	− 0.40			*t*(93)-0.05	0.96	− 0.01
ERT	60.35	25.75				66.74	22.68			
SRT	69.96	21.52				66.97	22.60			

Note. PCS = Physical Component Summary score. MSC = Mental Component Summary score. Both scores with range of 0–100

**Table 3:** ANOVA means and standard deviations for genotype and physical (PCS) and mental (MCS) HRQoL

Genotype	PCS		MCS	
	M	SD	M	SD
N370S/N370S	64.18	25.73	71.01	22.34
N370S/L444P	59.19	25.08	62.34	24.73
All Other	66.00	23.79	61.92	23.69

Note. PCS = Physical Component Summary score. MSC = Mental Component Summary score. Both scores with range of 0-100

### Pain and fatigue by sex and genotype

Table 4 indicates that females (*M* = 52.63, *SD* = 9.67) reported higher pain scores than males (*M* = 48.82, *SD* = 8.06), although this difference was not statistically significant, *t*(87) = -1.72, *p* = .09, *d* = − 0.41. Similarly, females (*M* = 54.00, *SD* = 10.46) reported higher fatigue scores than males (*M* = 51.45, *SD* = 12.25); however, this difference was also not statistically significant, *t*(87) = -0.97, *p* = .33, *d* = − 0.23.

**Table 4:** Independent samples t-test, means and standard deviations for symptoms of pain and fatigue by sex (*N* = 89)

Symptom	Sex				t(87)	*p*	Mean difference	Cohen’s d
	Males (*n* = 24)		Females (*n* = 65)					
	M	SD	M	SD				
Pain	48.82	8.06	52.63	9.67	-1.72	0.09	-1.72	− 0.41
Fatigue	51.45	12.25	54.00	10.46	-0.97	0.33	− 0.97	− 0.23

Note. Pain and Fatigue scores with possible T-score ranges of 40.70–77.00 and 33.10–77.70 respectively

Table 5 shows that adults with N370S/L444P genotype report higher pain and fatigue than other genotypes, although these results were not significant: Pain (*F*[2, 74] = 0.58, *p* = .561, η² = 0.02), Fatigue (*F*[2, 74] = 1.04, *p* = .358, η² = 0.03).

**Table 5:** ANOVA means and standard deviations for genotype and pain and fatigue T-scores

	Genotype	*n*	M	SD	F	df	*P*	η²
Pain	N370S/N370S	38	51.41	9.20	0.58	2, 74	0.561	0.02
	N370S/L444P	18	54.30	9.92				
	Other	21	51.80	9.93				
Fatigue	N370S/N370S	38	51.82	10.92	1.04	2, 74	0.358	0.03
	N370S/L444P	18	56.48	11.16				
	Other	21	53.32	12.08				

Note. Pain and Fatigue scores with T-score ranges of 40.70–77.00 and 33.10–77.70 respectively from this study

### Association of pain and fatigue with health-related quality of life

Pearson product moment correlations demonstrated a negative correlation between pain and PCS (*r* = − .83, *n* = 96, *p* < .001) with higher levels of pain associated with lower PCS scores. There was also a strong negative correlation between pain and MCS (*r* = − .69, *n* = 96, *p* < .001) with higher levels of pain associated with lower MCS. Additionally, there were strong negative correlations between fatigue and the PCS (*r* = − .68, *n* = 95, *p* < .001) and MCS (*r* = − .75, *n* = 95, *p* < .001).

### Predictors of health-related quality of life

Multiple regression was used to test six independent variables (age, treatment type, length on current treatment, treatment satisfaction, fatigue, and pain) as predictors of physical (PCS) and mental (MCS) HRQoL. For PCS (Table 6), the model accounted for 73% of the variance in the physical component of HRQoL *F*(6,77) = 39.07, *p* < .001, *R*^2^ = 0.75, Adjusted R^2^ = 0.73, 95% *CI* [152.95–204.91]. Age, fatigue, and pain were the only significant predictors of physical HRQoL: *p* = .01, *p* = .04, and *p* < .001 respectively. Semipartial correlations coefficients (*sr*) were squared to identify the change in *R*^*2*^. Age (*sr* = − 0.14) accounted for 2% of the variance in PCS, fatigue scores (*sr* = − 0.12) accounted for only 1%, while pain (*sr* = − 0.46) accounted for 21% of the variance. Length of time on current treatment was not a significant predictor of either physical or mental HRQoL, suggesting that persistent symptom burden may occur despite long-term treatment.

For MCS (Table 7), the model accounted for 59% of the variance in the mental component of HRQoL (*F*(6,77) = 21.09, *p* < .001, *R*^2^ = 0.62, Adjusted R^2^ = 0.59, 95% *CI* [109.08–167.53]). Fatigue and pain were the only significant predictors of mental HRQoL: *p* < .001 and *p* = .01 respectively. The squared semi-partial correlations coefficients (*sr*) indicated that fatigue (*sr* = − 0.32) accounted for 10% of the variance while pain (*sr* = − 0.20) accounted for only 4% of the variance in MCS.

Length of time on current treatment and treatment satisfaction were not significant predictors of either physical or mental HRQoL, suggesting that persistent symptom burden may occur despite long-term treatment and overall satisfaction with therapy.

**Table 6:** Regression coefficients of associations of 6 independent variables with PCS

Variable		β	SE	T	*p*	95% CI	sr
Age		− 0.15	0.09	-2.51	0.01	[-0.40, − 0.05]	− 0.14
Treatment type		0.12	3.64	1.74	0.09	[-0.93, 13.55]	0.10
Length current treatment		0.00	0.93	0.01	0.99	[-1.83, 1.86]	0.00
Treatment satisfaction		0.08	0.08	1.30	0.20	[-0.05, 0.25]	0.07
Fatigue		− 0.19	0.19	-2.13	0.04	[-0.79, -0.03]	− 0.12
Pain		− 0.68	0.22	-8.09	< 0.001	[-2.20, -1.33]	− 0.46
Constant							
*R* ^2^	0.75						
*Adj R* ^*2*^	0.73						
*F*-ratio	39.07 *p* < .001						
SEE	12.72						

Note: *β* = standardized coefficient; *SE* = standard error; *sr* = semipartial correlation coefficient; *R*^2^ = coefficient of determination; *AdjR*^*2*^ = adjusted coefficient of determination; SEE = standard error of the estimate

**Table 7:** Regression coefficients of associations of 6 independent variables with MCS

Variable		β	SE	T	*p*	95% CI		Sr
Age		0.06	0.10	0.75	0.45	− 0.12, 0.28		0.05
Treatment type		0.04	4.09	0.44	0.66	[-6.36, 9.93]		0.03
Length current treatment		0.04	1.04	0.46	0.65	[-1.60, 2.55]		0.03
Treatment satisfaction		0.11	0.09	1.45	0.15	[-0.05, 0.30]		0.10
Fatigue		− 0.49	0.22	-4.50	< 0.001	[-1.40, − 0.54]		− 0.32
Pain		− 0.29	0.25	-2.82	0.01	[-1.18, − 0.20]		− 0.20
Constant								
*R* ^2^	0.62							
*Adj R* ^*2*^	0.59							
*F*-ratio	21.09 *p*<.001							
SEE	14.30							

Note: *β* = standardized coefficient; *SE* = standard error; *sr* = semi partial correlation coefficient; *R*^2^ = coefficient of determination; *AdjR*^*2*^ = adjusted coefficient of determination; SEE = standard error of the estimate

### Comparison of SF-36 scores with U.S. population norms

Due to the significant results that indicate pain and fatigue are important contributors to HRQoL, an independent samples t-test was conducted to compare U.S. population normative values from the eight subscales of the SF-36, which make up the PCS and MCS summary measures, with the scores from this sample (Table 8). For seven of the eight SF-36 subscales, people with GD1 in this study reported significantly lower HRQoL than U.S. population norms.

**Table 8:** Comparison of SF-36 subscale scores for general US population normative values with people with GD1 in this study

SF-36 Subscale	U.S. Population Norms *N* = 2,474		People with GD1 in this study *N* = 97			
	Mean	SD	Mean	SD	Mean Difference	Significance
Physical functioning^1^	84.52	22.89	72.38	28.01	11.77	< 0.001
Physical role^1^	81.19	33.80	63.06	41.30	18.13	< 0.001
Bodily pain^1^	75.49	23.56	65.85	24.70	9.64	< 0.001
General health^1^	72.21	20.17	52.63	22.89	19.58	< 0.001
Vitality^2^	61.05	20.87	49.02	23.88	12.03	< 0.001
Social functioning^2^	83.60	22.38	74.61	25.54	8.99	< 0.001
Emotional role^2^	81.29	33.03	73.88	37.65	7.41	0.06
Mental health^2^	74.84	18.01	70.49	18.60	4.35	0.03

Note. U.S. Population Normative values from “SF-36 Physical & Mental Health Summary

Scales: A User’s Manual,” by J. E. Ware, 1994. Copyright 1994 by John E. Ware, Jr., Ph.D

^1^ Subscales comprising the Physical Component Summary score (PCS) of HRQoL

^2^ Subscales comprising the Mental Component Summary score (MCS) of HRQoL

## Discussion

This cross-sectional study is the first to examine whether individual characteristics, treatment type, treatment satisfaction, and symptoms of pain and fatigue are associated with HRQoL among adults with GD1 receiving either ERT or SRT. Strong correlations were found between pain, fatigue and HRQoL. Pain, fatigue, and age predicted physical HRQoL, while pain and fatigue predicted mental HRQoL.

Pain and fatigue are common in GD1, despite long-standing treatment availability. Symptom burden and HRQoL impairment were not associated with duration of treatment or treatment satisfaction, suggesting that prolonged therapy and overall satisfaction with treatment may not fully resolve persistent pain and fatigue in some individuals with GD1. Delays in diagnosis and treatment initiation may contribute to persistent symptom burden in some individuals with GD1. Prior studies have shown that delayed recognition and treatment are associated with progression to irreversible disease manifestations, including avascular necrosis, chronic bone pain, pathologic fractures, liver disease, and other complications that may continue to affect quality of life despite subsequent therapy [8, 17]. Early diagnosis and timely initiation of disease-specific treatment are therefore important in reducing long-term morbidity. Although treatment reduces pain, pain continues to affect HRQoL [24, 26, 30, 46]. Fatigue similarly improves with ERT or SRT but remains prevalent among treated individuals [9, 22]. Limited research has examined how fatigue relates to HRQoL in GD1; this study helps address that gap. Findings demonstrated a strong negative association between both pain and fatigue and HRQoL. Individuals reporting higher levels of these symptoms had lower physical and mental HRQoL. Overall HRQoL among people with GD1 was significantly reduced compared to the U.S. normative values, with pain and fatigue likely major contributors. Importantly, these findings suggest a potential disconnect between improvements in traditional clinical markers and persistent patient-reported symptom burden and indicate a need for further research. Even in the context of long-term therapy, pain and fatigue appear to be dominant drivers of impaired HRQoL, suggesting that current treatments may not fully address the overall disease experience.

Females in this study reported lower HRQoL and higher pain and fatigue levels than males, although differences were not statistically significant—likely due to the small sample size common in rare disease research. This aligns with previous work documenting lower HRQoL among females with GD1 compared with males [20, 26]. Higher symptom burden among females may contribute to poorer overall HRQoL.

Consistent with earlier studies [2, 24, 27–28], individuals with the N370S/N370S genotype in this study reported higher HRQoL and lower pain and fatigue relative to those with heterozygous genotypes. Although these differences did not reach significance, again likely due to sample size, the findings are directionally consistent with known genotype and phenotype correlations reported in the GD1 literature.

Given that pain and fatigue were significant predictors of HRQoL, clinicians should clinicians should continue to routinely assess pain, fatigue, and HRQoL using standardized patient-reported outcome measures as part of comprehensive GD1 care. Development and use of GD1-specific validated measures for pain and fatigue could enhance understanding of disease-related symptom experiences. Treatment decisions should incorporate shared goal setting between individuals with GD1 and providers at each visit. Clinical guidelines for GD1 should potentially include strategies for addressing pain and fatigue including pharmacologic treatments, alternative medical systems, biologically based therapies, manipulative and body-based methods, and mind-body medicine, some of which have shown benefits for functional status and HRQoL [47] and should be an area of future research. Aging was also a significant predictor of physical HRQoL; older adults with GD1 may have accumulated irreversible manifestations prior to treatment availability. Thus, aging-related assessments should be incorporated into clinical care. Clinicians should closely monitor clinical status as well as pain, fatigue, and both physical and mental HRQoL over time. Additionally, future studies should examine the impact of diagnostic delay on long-term symptom burden and HRQoL among individuals with GD1, as delayed diagnosis may contribute to irreversible disease manifestations and persistent pain and fatigue.

Although differences in sex and genotype were observed across pain, fatigue, and HRQoL, none reached statistical significance, likely due to limited sample size. Further research is needed to clarify these patterns, particularly given that females in the general U.S. population report lower HRQoL than males [42]. Additional studies should explore sex-specific clinical biomarkers, therapeutic dose optimization, and the impact of comorbid conditions. For individuals with theN370S/L444P genotype, future research should investigate potential opportunities for optimizing ERT or SRT dosing and identifying relevant biomarkers.

### Limitations

This study had a small number of participants, as is typical in rare disease research, which may limit statistical power and generalizability. Participants may have had prior exposure to the questionnaires used, resulting in potential response bias. In addition, recruitment through online support groups may introduce selection bias, as individuals who are active online may differ from those who are not. Finally, the cross-sectional design precludes inference of causality between pain, fatigue, and HRQoL.

## Conclusions

Patient-reported outcomes provide essential insight beyond traditional clinical measures and are critical to monitoring Gaucher disease. Despite long-term treatment, people with GD1 continue to report pain and fatigue, both of which significantly affect physical and mental health-related quality of life (HRQoL).

Development of validated GD1-specific measures of pain and fatigue may further enhance routine clinical assessment and disease monitoring. Additional research is needed to evaluate targeted and complementary approaches to reduce symptom burden and improve HRQoL. Clinicians should also recognize that individual factors, including treatment type, sex, and genotype, may influence symptom burden and patient experience.

Future research should explore these variables more closely to better understand their contributions and guide more personalized approaches to care. Taken together, these findings underscore the importance of integrating patient-reported outcomes into routine care to more fully capture disease burden and improve patient-centered outcomes in GD1.

## Data Availability

The datasets used and/or analyzed during the current study are available from the corresponding author on reasonable request.

## Abbreviations

HRQoL: Health-related quality of life
GD1: Gaucher disease type 1
ERT: Enzyme replacement therapy
SRT: Substrate reduction therapy
PCS: Physical component summary
MCS: Mental Component summary

## References

[1] Stirnemann J, Belmatoug N, Camou F, Serratrice C, Froissart R, Caillaud C, et al. A review of Gaucher disease pathophysiology, clinical presentation and treatments. Int J Mol Sci. 2017;18:441. 10.3390/ijms18020441. 28218669 PMC5343975

[2] Mistry PK, Cappellini MD, Lukina E, Özsan H, Pascual SM, Rosenbaum H, et al. A reappraisal of Gaucher disease: diagnosis and disease management algorithms. Am J Hematol. 2010;86(1):110–5. 10.1002/ajh.21888 PMC3058841 21080341

[3] Nalysnyk L, Rotella P, Simeone JC, Hamed A, Weinreb N. Gaucher disease epidemiology and natural history: a comprehensive review of the literature. Hematology. 2017;22(2):65–73. 10.1080/10245332.2016.1240391. 27762169

[4] Van Rossum A, Holsopple M. Enzyme replacement or substrate reduction? A review of Gaucher disease treatment options. Hosp Pharm. 2016;51(7):553–63. 10.1310/hpj5107-553. 27559188 PMC4981103

[5] Balwani M, Burrow TA, Charrow J, Goker-Alpan O, Kaplan P, Kishnani PS, et al. Recommendations for the use of eliglustat in the treatment of adults with Gaucher disease type 1 in the United States. Mol Genet Metab. 2016;117:95–103. 10.1016/j.ymgme.2015.09.002. 26387627

[6] Devigili G, De Filippo M, Ciana G, Dardis A, Lettieri C, Rinaldo S, et al. Chronic pain in Gaucher disease: skeletal or neuropathic origin? Orphanet J Rare Dis. 2017;12:148. 10.1186/s13023-017-0700-7. 28859662 PMC5580212

[7] Marcucci G, Zimran A, Bembi B, Kanis J, Reginster JY, Rizzoli R, et al. Gaucher disease and bone manifestations. Calcif Tissue Int. 2014;95:477–94. 10.1007/s00223-014-9923-y. 25377906

[8] Drelichman G, Escobar NF, Basack N, et al. Skeletal involvement in Gaucher disease: an observational multicenter study of prognostic factors in Argentine patients. Am J Hematol. 2016;91(10):E449–53. 10.1002/ajh.24486. 27420181

[9] Chen ZY, Pappadopulos E, Wajnrajch M, et al. Rethinking fatigue in Gaucher disease. Orphanet J Rare Dis. 2016;11:53. 10.1186/s13023-016-0435-x. 27129405 PMC4850725

[10] Hexner-Erlichman Z, Rosenbaum H. Fatigue in Gaucher disease, a key quality-of-life concern: A case series. Ann Clin Case Rep. 2024;9:2644. 10.25107/2474-1655.2644.

[11] Serfecz JC, Saadin A, Santiago CP, Zhang Y, Bentzen SM, Vogel SN, Feldman RA. C5a activates a pro-inflammatory gene expression profile in human Gaucher iPSC-derived macrophages. Int J Mol Sci. 2021;22(18):9912. 10.3390/ijms22189912. 34576075 PMC8466165

[12] Bennett LL, Turcotte K. Eliglustat tartrate for the treatment of adults with type 1 Gaucher disease. Drug Des Devel Ther. 2015;9:4639–47. 10.2147/DDDT.S77760. 26345314 PMC4554398

[13] Dandana A, Khelifa SB, Chahed H, Miled A. Gaucher disease: clinical, biological and therapeutic aspects. Pathobiology. 2016;83:13–23. 10.1159/000440865. 26588331

[14] Gary SE, Ryan E, Steward AM, Sidransky E. Recent advances in the diagnosis and management of Gaucher disease. Expert Rev Endocrinol Metab. 2018;13(2):107–18. 10.1080/17446651.2018.1445524. 30058864 PMC6129380

[15] Camou F, Lagadec A, Coutinho A, Berger ML, Cador-Rousseau B, Gaches F, Belmatoug N. Patient-reported outcomes of patients with Gaucher disease type 1 treated with eliglustat in real-world settings: the ELIPRO study. Mol Genet Metab. 2023;140(3):107667. 10.1016/j.ymgme.2023.107667. 37597334

[16] Elstein D, Belmatoug N, Bembi B, Deegan P, Fernandez-Sasso D, Giraldo P, et al. Twelve years of the Gaucher outcomes survey (GOS): insights, achievements, and lessons learned from a global patient registry. J Clin Med. 2024;13:3588. 10.3390/jcm13123588. 38930117 PMC11204885

[17] Goker-Alpan O. Therapeutic approaches to bone pathology in Gaucher disease: past, present and future. Mol Genet Metab. 2011;104:438–47. 10.1016/j.ymgme.2011.08.004. 21889384

[18] Damiano AM, Pastores GM, Ware JE Jr. The health-related quality of life of adults with Gaucher’s disease receiving enzyme replacement therapy. Qual Life Res. 1998;7:373–86. 9691718 10.1023/a:1008814105603

[19] Elstein D, Abrahamov A, Oz A, Arbel N, Baris H, Zimran A. Home therapy infusions with velaglucerase alfa. Blood Cells Mol Dis. 2015;55:415–8. 10.1016/j.bcmd.2015.09.002. 26460268

[20] Giraldo P, Solano V, Perez-Calvo JI, Giralt M, Rubio-Felix D. Quality of life related to type 1 Gaucher disease: Spanish experience. Qual Life Res. 2005;14:453–61. 15892434 10.1007/s11136-004-0794-y

[21] Hayes RP, Grinzaid KA, Duffey EB, Elsas LJII. The impact of Gaucher disease and its treatment on quality of life. Qual Life Res. 1998;7:521–34. 9737142 10.1023/a:1008878425167

[22] Lukina E, Watman N, Dragosky M, Lau H, Arreguin EA, Rosenbaum H, et al. Outcomes after 8 years of eliglustat therapy for Gaucher disease type 1. Am J Hematol. 2019;94:29–38. 10.1002/ajh.25300. 30264864 PMC6587500

[23] Masek BJ, Sims KB, Bove CM, Korson MS, Short P, Norman DK. Quality of life assessment in adults with type 1 Gaucher disease. Qual Life Res. 1999;8:263–8. 10472157 10.1023/a:1008859420641

[24] Weinreb N, Barranger J, Packman S, Prakash-Cheng A, Rosenbloom B, Sims K, et al. Imiglucerase improves quality of life in patients with skeletal manifestations of Gaucher disease. Clin Genet. 2007;71:576–88. 10.1111/j.1399-0004.2007.00811.x. 17539908

[25] Alioto AG, Gomez R, Moses J, Paternostro J, Packman S, Packman W. Quality of life and psychological functioning of pediatric and young adult patients with Gaucher disease type 1. Am J Med Genet A. 2020;182A:1130–42. 10.1002/ajmg.a.61533. 32125090

[26] Qi X, Xu J, Shan L, Li Y, Cui Y, Liu H, Wang K, Gao L, Kang Z, Wu Q. Economic burden and health-related quality of life of ultra-rare Gaucher disease in China. Orphanet J Rare Dis. 2021;16:358. 10.1186/s13023-021-01963-6. 34380529 PMC8356434

[27] Weinreb NJ, Finegold DN, Feingold E, Zeng Z, Rosenbloom BE, Shankar SP, Amato D. Evaluation of disease burden and response to treatment using DS3. Orphanet J Rare Dis. 2015;10:64. 10.1186/s13023-015-0280-3. 25994334 PMC4471923

[28] Zimran A, Kay A, Gelbart T, Garver P, Thurston D, Saven A, Beutler E. Gaucher disease: clinical features of 53 patients. Med (Baltim). 1992;71(6):337–53. 1435229

[29] Oliveira FL, Alegra T, Dornelles A, Krug BC, Netto CBO, Sica da Rocha N, Picon PD. Quality of life of Brazilian patients with Gaucher disease and Fabry disease. JIMD Rep. 2012;7:31–7. 10.1007/8904_2012_136. 23430492 PMC3573176

[30] Weinreb NJ, Aggio MC, Andersson HC, et al. Gaucher disease type 1: revised recommendations. Semin Hematol. 2004;41(Suppl 5):15–22. 10.1053/j.seminhematol.2004.07.010. 15468046

[31] Weinreb NJ, Goldblatt J, Villalobos J, Charrow J, Cole JA, Kerstenetzky M, vom Dahl S, Hollak C. Long-term clinical outcomes after 10 years of imiglucerase treatment. J Inherit Metab Dis. 2013;36:543–53. 10.1007/s10545-012-9528-4. 22976765 PMC3648688

[32] Mistry PK, Charrow J, Cox T, Lukina E, Marinakis T, Foster MC, Gaemers SJM, Peterschmitt J. Long-term effects of oral eliglustat on skeletal manifestations. Blood. 2018;132(Suppl 1):2396. 10.1182/blood-2018-99-117289.

[33] Andersson HC, Charrow J, Kaplan P, Mistry P, Pastores GM, Prakesh-Cheng A, Rosenbloom BE, Scott CR, Wappner RS, Weinreb NJ. Individualization of long-term enzyme replacement therapy. Genet Med. 2005;7(2):105–10. 10.1097/01.GIM.0000153660.88672.3C. 15714077

[34] Mistry PK, Lukina E, Ben Turkia H, et al. Clinical outcomes after 4.5 years of eliglustat therapy for Gaucher disease type 1: Phase 3 ENGAGE trial final results. Am J Hematol. 2021;96(9):1156–65. 10.1002/ajh.26276. 34161616 PMC8457136

[35] Serrano-Gonzalo I, Irun P, Alfonso P, Giraldo P. Real-world effectiveness and safety of eliglustat. Orphanet J Rare Dis. 2023;18:262. 10.1186/s13023-023-02939-4.37658423

[36] Stevenson DA, Carey JC. Health-related quality of life measures in genetic disorders. Am J Med Genet C Semin Med Genet. 2009;151 C:255–60. 19621444 10.1002/ajmg.c.30217 PMC2745208

[37] Harris PA, Taylor R, Thielke R, Payne J, Gonzalez N, Conde JG. Research electronic data capture (REDCap). J Biomed Inf. 2009;42(2):377–81. 10.1016/j.jbi.2008.08.010. PMC2700030 18929686

[38] Harris PA, Taylor R, Minor BL, et al. The REDCap consortium. J Biomed Inf. 2019;95:103208. 10.1016/j.jbi.2019.103208. PMC7254481 31078660

[39] Ware JE Jr, Sherbourne CD. The MOS 36-item short-form health survey (SF-36). Med Care. 1992;30(6):473–83. 1593914

[40] McHorney CA, Ware JE Jr, Raczek AE. The MOS 36-item short-form health survey (SF-36): II. Psychometric and clinical tests of validity in measuring physical and mental health constructs. Med Care. 1993;31(3):247–63. 8450681 10.1097/00005650-199303000-00006

[41] Brazier JE, Harper R, Jones NM, O’Cathain A, Thomas KJ, Usherwood T, Westlake L. Validating the SF-36 health survey questionnaire: new outcome measure for primary care. BMJ. 1992;305(6846):160–4. 10.1136/bmj.305.6846.160. 1285753 PMC1883187

[42] Ware JE Jr, Kosinski M, Keller SD. SF-36 physical and mental health summary scales: a user’s manual. Boston: Health Assessment Lab; 1994.

[43] Atkinson MJ, Sinha A, Hass SL, Colman SS, Kumar RN, Brod M, Rowland CR. Validation of TSQM. Health Qual Life Outcomes. 2004;2:12. 10.1186/1477-7525-2-12. 14987333 PMC398419

[44] Amtmann D, Cook KF, Jensen MP, et al. Development of PROMIS fatigue item bank. Pain. 2010;150:173–82. 10.1016/j.pain.2010.04.025. 20554116 PMC2916053

[45] Faul F, Erdfelder E, Lang AG, Buchner A. G*Power 3. Behav Res Methods. 2007;39(2):175–91. 10.3758/BF03193146. 17695343

[46] Weinreb NJ, Camelo JS, Charrow J, McClain MR, Mistry P, Belmatoug N. ICGG Gaucher Registry (NCT00358943) investigators. Gaucher disease type 1 patients from the ICGG Gaucher Registry sustain initial clinical improvements during twenty years of imiglucerase treatment. Mol Genet Metab. 2021;132:100–11. 10.1016/j.ymgme.2020.12.295. 33485799

[47] Nguyen HT, Grzywacz JG, Lang W, Walkup M, Arcury TA. Effects of complementary therapy. J Altern Complement Med. 2010;16(7):701–6. 10.1089/acm.2009.0442. 20590482 PMC3110827

